# Metformin promotes angiogenesis by enhancing VEGFa secretion by adipose-derived stem cells via the autophagy pathway

**DOI:** 10.1093/rb/rbad043

**Published:** 2023-04-24

**Authors:** Zihan Tao, Lei Liu, Minliang Wu, Qianqian Wang, Yuchong Wang, Jiachao Xiong, Chunyu Xue

**Affiliations:** Department of Plastic Surgery, Changhai Hospital, Naval Medical University, Shanghai, China; Department of Plastic Surgery, Changhai Hospital, Naval Medical University, Shanghai, China; Department of Plastic Surgery, Changhai Hospital, Naval Medical University, Shanghai, China; Department of Marine Biomedicine and Polar Medicine, Naval Special Medical Center, Naval Medical University, Shanghai, China; Department of Plastic Surgery, Changhai Hospital, Naval Medical University, Shanghai, China; School of Life Sciences and Technology, Tongji University, Shanghai, China; Department of Plastic and Reconstructive Surgery, School of Medicine, Shanghai East Hospital, Tongji University, Shanghai, China; Department of Plastic Surgery, Changhai Hospital, Naval Medical University, Shanghai, China

**Keywords:** metformin, stem cell, angiogenesis, wound healing, human adipose tissue-derived stem cell

## Abstract

Human adipose tissue-derived stem cell (ADSC) derivatives are cell-free, with low immunogenicity and no potential tumourigenicity, making them ideal for aiding wound healing. However, variable quality has impeded their clinical application. Metformin (MET) is a 5′ adenosine monophosphate-activated protein kinase activator associated with autophagic activation. In this study, we assessed the potential applicability and underlying mechanisms of MET-treated ADSC derivatives in enhancing angiogenesis. We employed various scientific techniques to evaluate the influence of MET on ADSC, assess angiogenesis and autophagy in MET-treated ADSC *in vitro*, and examine whether MET-treated ADSC increase angiogenesis. We found that low MET concentrations exerted no appreciable effect on ADSC proliferation. However, MET was observed to enhance the angiogenic capacity and autophagy of ADSC. MET-induced autophagy was associated with increased vascular endothelial growth factor A production and release, which contributed to promoting the therapeutic efficacy of ADSC. *In vivo* experiments confirmed that in contrast to untreated ADSC, MET-treated ADSC promoted angiogenesis. Our findings thus indicate that the application of MET-treated ADSC would be an effective approach to accelerate wound healing by promoting angiogenesis at wound sites.

## Introduction

Non-healing cutaneous wounds represent a crucial global healthcare and societal challenge. Morbidity from diabetes is a major factor for persistent, non-healing wounds that put patients in danger of limb amputation [1, 2]. The diminished ability to rebuild microvasculature via angiogenesis is the primary pathophysiological cause of non-healing wounds in peripheral vascular disease and diabetes [3]. Angiogenesis is required for the transfer of oxygen, nutrients, and other essential factors into proximal injured regions during the entire tissue regeneration process in all types of cutaneous wound healing [4, 5]. Additionally, a range of procedures and dressings have been used to promote chronic wound healing, including growth factor delivery, stem cell therapies, gene therapies, and mechanical-based stimulation [6–10].

**Figure:**
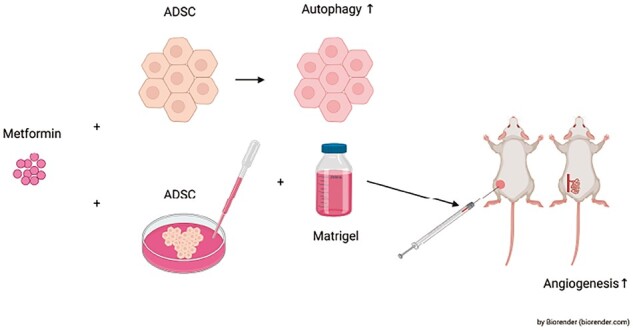


Previous research has demonstrated that human adipose tissue-derived stem cells (ADSC), a population of pluripotent mesenchymal cells, are effective for wound healing, as they convey a range of cytokines and growth factors, and influence the biological processes of skin cells implicated in the wound healing process, including fibroblasts, keratinocytes, and endothelial cells. Given the abundance of ADSC sources, they are considered highly advantageous for therapeutic use and are extensively utilized in the field of regenerative medicine [11]. The key variables that contribute to the biological effects of ADSC are paracrine cytokines, exosomes, and other active compounds [12–16]. Interestingly, as alternatives to traditional ADSC therapy, ADSC-conditioned medium (ADSC-CM) and ADSC-exosomes (ADSC-Exos) have recently received considerable research attention. ADSC-CM can stimulate tissue healing and control immunity by harbouring active molecules released by ADSC, including cytokines, exosomes, DNA, and RNA [17]. Although ADSC-CM contains high concentrations of vascular endothelial growth factor A (VEGFa) and hepatocyte growth factor [18], cytokine generation in ADSC-CM is typically variable, thereby limiting its therapeutic utility. In this regard, certain chemicals have been established to stimulate the release of active compounds secreted by mesenchymal stem cells (MSC), some of which can also effectively enhance the release of extracellular vesicles [19–22]. Consequently, chemical methods may be a promising approach to enhance the release of active substances secreted by ADSC. In this study, we accordingly sought to stimulate ADSC with a chemical that would contribute to promoting cytokine secretion, which would be beneficial with respect to wound vascularization.

Metformin (MET), a biguanide, is a routinely employed oral antihyperglycemic medication used to treat type 2 diabetes mellitus [23], the therapeutic activity of which is mediated via a range of mechanisms, including the inhibition of glucose production in the liver. MET is an insulin booster that enhances insulin sensitivity [24], and consequently, it may be beneficial from the perspective of wound healing, as insulin resistance has been shown to impede the healing of excisional skin wounds by delaying contraction and re-epithelialization [25]. Importantly, MET has been reported to stimulate autophagy [26] and may also influence MSC secretion.

Autophagy is a cellular process that by selectively degrading dysfunctional organelles and intracellular pathogens, promotes cell survival during both growth and conditions of starvation. These mechanisms serve as protective measures for the cell, ensuring correct cellular function and preventing the accumulation of potentially harmful materials [27–29]. Autophagy protects MSC from apoptosis in an inflammatory microenvironment or under hypoxic conditions via the 5′ adenosine monophosphate-activated protein kinase (AMPK)/mammalian target of rapamycin (mTOR) pathway [30, 31]. Moreover, autophagy is involved in neurogenesis [32] and osteogenesis [33], and also plays an important role in angiogenesis during ischaemic tissue regeneration by inhibiting apoptosis of endothelial cells under hypoxia, thereby enhancing their survival and promoting their proliferation and differentiation [34]. Autophagy has also been demonstrated to be involved in VEGFa-mediated angiogenesis relevant to endothelial cells [35]. However, it remains unclear as to whether the autophagy of ADSC has a therapeutic effect on wound healing. Consequently, we sought to investigate whether, by promoting autophagy, MET can promote cytokine secretion of ADSC, which is essential for the application of ADSC-CM for wound healing.

Our objective in this study was to characterize the influence of MET with respect to enhancing the angiogenic capacity of ADSC-CM. To this end, we investigated the stimulatory effect of MET on ADSC and focussed on whether MET-enhanced autophagy in ADSC is associated with ADSC-related angiogenesis. To assess the changes in cytokine secretion induced by MET simulation, we further compared the angiogenic potential of ADSC-CM with or without treatment with MET *in vivo* and *in vitro*. Our results revealed that the application of MET-treated ADSC would be a potentially effective approach for accelerating wound healing by promoting angiogenesis at the site of wounding.

## Materials and methods

### Ethical approval

The collection and utilization of human ADSC from adipose tissue were approved by the Biomedical Research Ethics Committee of Naval Medical University in Shanghai, China, according to the guidelines of the Declaration of Helsinki. Adipose tissues were procured from three medically fit individuals between the ages of 25 and 45 years, having initially obtained their written informed consent.

### Cell isolation and culture

Adipose tissues were digested for 60 min with collagenase type I, this process being terminated by the addition of OriCell Human ADSC Complete Medium (HUXMD-90011; Cyagen Biosciences, Santa Clara, CA, USA). The pellet comprising cell debris was collected via centrifugation at 100 × *g* for 5 min. The cells were cultured under humidified conditions in a 5% CO_2_ atmosphere at 37°C, and the medium was refreshed at 72-h intervals. The cells were defined as passage 1 having reached 80% confluence, and were sequentially re-plated until the fourth passage for subsequent experiments. To induce differentiation, the passaged cells were subjected to a 2-week incubation in adipogenic differentiation medium (HUXMD-90021; Cyagen Biosciences), 3 weeks in osteogenic differentiation medium (HUXMD-90031; Cyagen Biosciences), or 3 weeks in chondrogenic differentiation medium (HUXMD-90041; Cyagen Biosciences). The induced cells were subsequently subjected to differentiation assays via staining with Oil Red O for 30 min to evaluate adipogenic differentiation, with Alizarin Red S (Cyagen, Guangzhou, China) for 5 min at room temperature to visualize osteogenic differentiation, or with Alcian Blue (Cyagen) for 1 h at 37°C to visualize chondrogenic differentiation. The stained cells were imaged following a washing step with phosphate-buffered saline (PBS) to eliminate any remaining dye (Supplementary Fig. S1a).

### Cell viability analysis

Cell viability was assessed using a Cell Counting Kit-8 assay (CCK-8; Yeasen, Shanghai, China), according to the manufacturer’s instructions. After seeding ADSC (5 × 10^3^/well) in a 96-well plate and treating appropriately, 10 μl of CCK-8 solution was added to each well. The samples were incubated for a period of 1.5 h, after which absorbances were measured at 450 nm using a microplate reader (Bio-Rad Laboratories, Hercules, CA, USA). Cell viability was determined by calculating the difference in optical density values obtained for the experimental and control groups.

### Flow cytometry

To prepare the cells for flow cytometry analysis, a single-cell suspension containing 1 × 10^6^ ADSC was prepared and subsequently fixed with 4% paraformaldehyde (Solarbio, Beijing, China). Following fixation, the cells were washed with PBS, and CD73 (PE; BD Biosciences, San Jose, CA, USA), CD90 (FITC; BD Biosciences), CD105 (APC; BD Biosciences), CD31 (APC; BD Biosciences), CD235a (PE; BD Biosciences), and CD45 (FITC; BD Biosciences) were used to label the cells. Flow cytometry was performed to determine the intensity of cell labelling.

### Cell cycle analysis

For the purposes of cell cycle analysis, 1 × 106 ADSC were collected and washed three times with PBS, and thereafter labelled with propidium iodide (Beyotime Biotechnology, Shanghai, China) according to the manufacturer’s instructions. The labelled cells were subjected to flow cytometry analysis, and the resulting data were analysed using ModFit software (version 3.1).

### Extract culture medium of ADSC

Hundred micrometres MET (24 h), 10 nM rapamycin (6 h), and 10 mM 3-MA (6 h) were used respectively to stimulate ADSC for increasing or decreasing autophagy [36]. After the stimulation, the cell supernatant containing the medicine mentioned before was removed, and fresh ADSC culture medium was added to treat ADSC, whose autophagy level has been changed, after 24 h, the MET-treated ADSC-CM, the rapamycin-treated ADSC-CM, the 3-MA-treated ADSC-CM, and the MET + 3-MA-treated ADSC-CM were extracted and centrifuged (2000 × *g* for 20 min).

### Cell scratch assay

The migratory capacity of cells was evaluated using human umbilical vein endothelial cells (HUVECs). HUVECs were seeded in six-well plate inserts. After the cells had reached confluence, they were subjected to a 16-h starvation treatment with serum deprivation. For the purposes of the scratch assay, a sterile pipette tip (200 μl) was used to create a straight line on the bottom well, and photographs were captured at 0-, 12-, and 24-h time points. ImageJ software was employed to assess the migratory capacity of cells. The experiment was repeated three times for validation.

### Tube formation assay

Initially, the wells of a 96-well plate were coated evenly with 60 μl of Matrigel (BD Biosciences), which was then polymerized for 30 min at 37°C. HUVECs were then seeded at a density of 2 × 10^4^ cells/well and incubated for 4–6 h with different groups of ADSC-CM. A phase-contrast microscope was used to observe tube formation, and photographs were taken periodically. Three fields were examined per test condition, and the total number of branching points, tube numbers, covered area (%), and total length of tubes (μm) were calculated using ImageJ software with an installed plugin (https://Image J.nih.gov/ij/plugins/index.html). The experiment was conducted in triplicate.

### Western blot analysis

Prior to treatment, the wells of six-well plates were seeded with 1 × 106 cells. A protease inhibitor-containing radioimmunoprecipitation assay lysis buffer (Beyotime Biotechnology, Shanghai, China) was used to lyse the cells. Proteins were separated using sodium dodecyl sulphate–polyacrylamide gel electrophoresis (Beyotime Biotechnology, Shanghai, China) and subsequently transferred to polyvinylidene fluoride membranes. After washing membranes for 15 min with a quick-blocking reagent (Beyotime Biotechnology, Shanghai, China), they were incubated overnight with a primary antibody VEGFa (1:500, Abcam, UK), Ang II (1:1000, Abcam, UK), LC3 I/II (1:1000, Abcam, UK), Beclin-1 (1:1000, CST, USA), Atg 5 (1:1000, CST, USA), or GAPDH (1:1000, Engibody, China). Horseradish peroxidase (HRP)-conjugated secondary antibodies (1:2000, Beyotime Biotechnology, Shanghai, China) were used to detect the bands using ECL Plus reagent (Bio-Rad Laboratories). The experiment was performed in triplicate.

### Enzyme-linked immunosorbent assay (ELISA) of VEGFa and interleukin (IL)-6

The different groups of ADSC-CMs were collected as described previously and concentrated using a concentration tube (MilliporeSigma, Burlington, MA, USA). Following centrifugation at 3400 × *g* for 40 min, the concentrated suspension of ADSC-CM was used for analysis. VEGFa and IL-6 levels were measured using commercially available ELISA kits (Elabscience Biotechnology Co., Ltd., Wuhan, China), in accordance with the manufacturer’s instructions. Using a microplate reader, we measured the absorbance of plates at 450 nm (Bio-Rad Laboratories). For each sample, we performed three independent experiments.

### Transmission electron microscopy (TEM)

ADSC were post-fixed overnight in 2.5% glutaraldehyde (Goodbio Technology, Wuhan, China), rinsed with PBS, and then fixed in 2% osmium acid for 1 h. Following a series of dehydration steps with ethanol, the ADSC were embedded in Epon resin. After staining for 1 h with uranyl acetate and lead citrate, the specimens were observed under a transmission electron microscope (FEI Tecnai G20 TWIN, USA), and images were obtained for random fields of view.

### Immunofluorescence analysis

Autophagy was assessed using an autophagy detection kit (ab139484; Abcam, Cambridge, UK), in accordance with the manufacturer’s protocol. ADSC were placed in the wells of a 96-well plate, treated with 100 μl of Microscope Dual Detection Reagent for 30 min at 37°C, and fixed for 20 min in 4% paraformaldehyde. Three independent researchers used a fluorescence microscope (Leica, Wetzlar, Germany) to capture images of autophagic vacuoles, and ImageJ software was used to calculate the fluorescence intensity of each image.

### Neutralizing VEGFa antibody assay

To eliminate the influence of the ADSC-secreted VEGFa on HUVECs, ADSC (1 × 10^6^/well) were seeded in the wells of a six-well plate, to which 5 μl/well VEGFa antibody (11066-R012; Sino Biological Inc., Beijing, China) was added. The culture supernatant of VEGFa antibody-treated ADSC was used for incubation of HUVECs at 37°C for 24 h, after which they were harvested and examined for further experimentation.

### *In vivo* Matrigel plug assay

The different groups of ADSC-CMs were collected as described previously and concentrated, following which the concentrated suspension was mixed with Matrigel (BD Biosciences). The Matrigel solution (200 μl) was subcutaneously injected into the dorsal side of the left and right flanks of mice. At the time of injection, the solution was at a temperature of 4°C and formed a plug as it warmed up to the body temperature (37°C). The mice were euthanized after 21 days, and the Matrigel plugs were removed. Depending on the amount of blood vessel ingrowth, the colour of the plugs ranged from white to red.

### Haematoxylin and eosin staining

Matrigel plugs were fixed for 12 h in 4% paraformaldehyde at 4°C, and after gradually dehydrating, the specimens were embedded in paraffin blocks and sliced into 4-μm-thick sections. Thereafter, they were then stained with haematoxylin and eosin (H&E) and observed and imaged under a light microscope.

### CD31 and NSE immunohistochemical staining

To quantify vascular formation in the Matrigel plug, we immunohistochemically examined the expression of CD31 and NSE. The samples were blocked with 5% BSA for 30 min, after which they were incubated overnight with a primary antibody. Following counterstaining the sections with haematoxylin and incubating with an HRP-conjugated secondary antibody, three different researchers used a microscope to observe and photograph the sections. The intensity of CD31 staining was assessed using the ImageJ software IHC Profiler plugin. The intensity with which endothelial cells were stained with CD31 was classified into following four categories: high positive (HP), positive (P), low positive (LP), and negative (N), with scores being calculated using the following formula:


CD31 staining intensity score=3HP+2P+1LP+0N

The scores thus obtained were subsequently subjected to statistical analysis. Furthermore, the maximum depth of endothelial cells (µm) was measured using ImageJ software to quantify angiogenesis.

### Terminal transferase-mediated DNA end labelling (TUNEL) assay

Trypsinized ADSC were resuspended in fresh medium at a final concentration of 1 × 10^5^ cells/ml, added to the wells of a 24-well plate at 400 μl/well, and cultivated for 24 h, after which they were fixed with 4% paraformaldehyde and rinsed with PBS. Thereafter, the cells were permeabilized with 0.1% Triton X100 in PBS for 2 min on ice. After washing with PBS, the cells were treated for 60 min at 37°C in the dark with 50 μl of TUNEL test solution, then sealed with an anti-fluorescence quenching solvent, and subsequently inspected using a fluorescence microscope (Leica).

### Statistical analysis

Data are presented as the means±SDs. All experiments were conducted independently in triplicate. For comparisons between two groups, we used Student’s *t*-tests, and for comparisons among different groups, we used either a one-way or two-way analysis of variance with Tukey’s *post hoc* test. The statistical significance was determined using GraphPad Prism 8 programme (La Jolla, CA, USA), with the statistically significant threshold set at *P* < 0.05 (*P < 0.05, **P < 0.01, ***P < 0.001).

## Results

### A low MET concentration has no effect on ADSC proliferation

**Figure 1.**
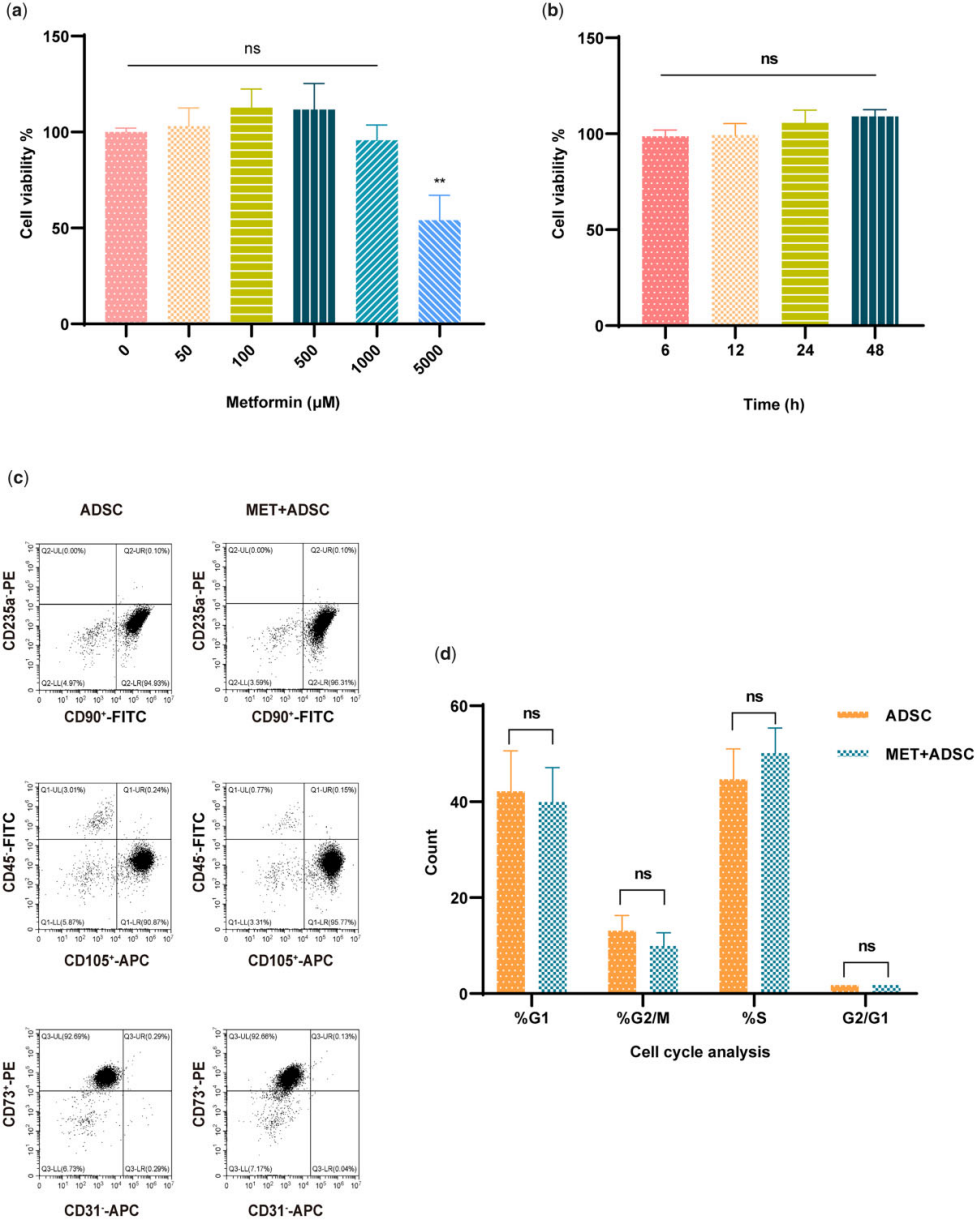
Effects of MET on the proliferation and surface markers of ADSC. (**a**) The effects of MET on ADSC proliferation. Using a microplate reader, the vitality of ADSC treated with different MET doses (0, 50, 100, 500, 1000 and 5000 μM) was assessed (^ns^*P*>0.05, **P < 0.01, *n*=3). (**b**) Proliferation experiment of the effects of MET on ADSC. ADSC were treated using 100 μM MET at different time points (6, 12, 24, and 48 h) (^ns^*P* > 0.05, *n*=3). (**c**) Flow cytometry analysis of the effects of MET on ADSC; ADSC were labelled using three antibody panels: 1. CD90^+^-FITC, CD235a—PE; 2. CD105^+^-APC, CD45—FITC; and 3. CD73^+^-PE, CD31—APC. (**d**) Cell cycle analysis of untreated and MET-treated ADSC (^ns^*P* > 0.05, *n*=3).

Cell proliferation tests were performed to determine the appropriate MET concentration for examining the influence of MET on ADSC proliferation. The results indicated that a low MET concentration (0–1000 μM) had no appreciable influence on ADSC proliferation (Fig. 1a). On the basis of these initial findings, an MET concentration 100 μM was determined to be the optimal concentration and was used in all following independent trials. The effect of MET treatment time was also examined using cell proliferation experiments, which revealed that 100 μM MET exerted no influence on ADSC proliferation within 48 h (Fig. 1b). Analysis of morphological data revealed that MET similarly had no discernible impact on the morphological traits of ADSC (Supplementary Fig. S1b). To determine the immunophenotype of the cells, we investigated the intensity of cell surface marker expression using flow cytometry. ADSC were labelled for flow cytometry using the following three antibody panels: 1. CD90^+^-FITC, CD235a—PE; 2. CD105^+^-APC, CD45—FITC; and 3. CD73^+^-PE, CD31—APC. We accordingly detected no significant difference between untreated and MET-treated ADSC with respect to the proportions of CD90^+^/CD235a^−^, CD105^+^/CD45^−^, and CD73^+^/CD31^−^ cells (Fig. 1c). Similarly, the results of a cell cycle study revealed no significant differences between the cell cycle patterns of MET-treated and untreated ADSC (Fig. 1d).

### MET enhances the angiogenic potential of ADSC

**Figure 2.**
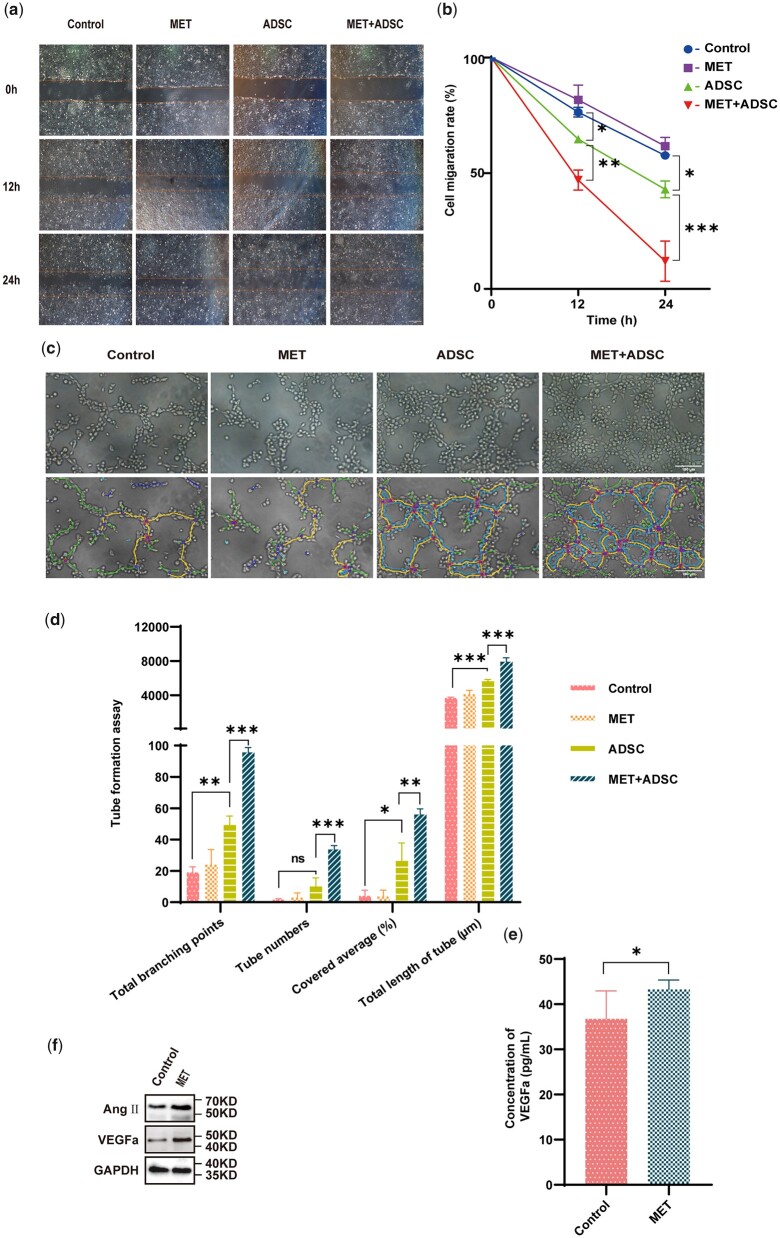
Effects of MET on the angiogenic capacity of ADSC *in vitro*. (**a**) The effect of MET-treated ADSC-CM on the migration of HUVECs. HUVECs were treated with MET (100 μM), untreated ADSC-CM, MET-treated ADSC-CM, or Dulbecco’s Modified Eagle’s Medium (DMEM) as the control (scale bar: 500 μm). (**b**) Estimation and demonstration of the ratio of healing following HUVEC injury is presented in a continuous line graph at 0, 12, and 24 h (*P<0.05, **P<0.01, ***P<0.001, *n*=3). (**c**) Angiogenic differences *in vitro* were evaluated using the *in vitro* tube formation of HUVECs. HUVECs were treated with MET (100 μM), untreated ADSC-CM, MET-treated ADSC-CM, or DMEM as the control at 6 h (scale bar: 100 μm). (**d**) Four following four indicators were assessed: total branching points, tube numbers, covered area (%), and total length tubes (μm) (^ns^*P*>0.05, *P<0.05, **P<0.01, ***P<0.001, *n*=3). (**e**) VEGFa secretion was assessed using MET-treated and untreated ADSC-CM via ELISA (*P<0.05, *n*=3). (**f**) Expression of angiogenesis-related proteins Ang II and VEGFa in MET-treated and untreated ADSC was analysed using western blotting.

We hypothesized that MET treatment of ADSC would promote an increase in the levels of VEGFa in ADSC-CM. Cell migration analyses revealed that ADSC-CM facilitated the migration of HUVECs (Fig. 2a). In addition, in response to MET treatment, ADSC-CM was observed to enhance this process, with a substantial difference being detected between measurements performed at 12 and 24 h (Fig. 2b). Furthermore, a tube formation assay performed *in vitro* revealed that ADSC-CM promoted tube formation in HUVECs, and MET significantly enhanced this effect *in vitro* (Fig. 2c). Furthermore, the HUVECs cultured in MET-treated ADSC-CM were characterized by increases in total branching points, tube numbers, covered area (%), and total length of tubes (μm) in Matrigel compared with those in the non-treated ADSC-CM group (Fig. 2d). Moreover, to investigate the molecular mechanisms underlying angiogenesis, we measured the concentration of VEGFa in ADSC-CM using ELISA. The results revealed that ADSC pre-treated with MET released larger amounts of VEGFa than untreated ADSC (Fig. 2e). We also examined protein expression in ADSC during angiogenesis. The expression of angiopoietin II (Ang II) and VEGFa proteins was observed to be higher in the MET-treated ADSC-CM group than in the untreated ADSC-CM group (Fig. 2f). These findings accordingly indicate that MET can enhance the angiogenic potential of ADSC by stimulating the secretion of VEGFa.

### MET increases the autophagy of ADSC

**Figure 3.**
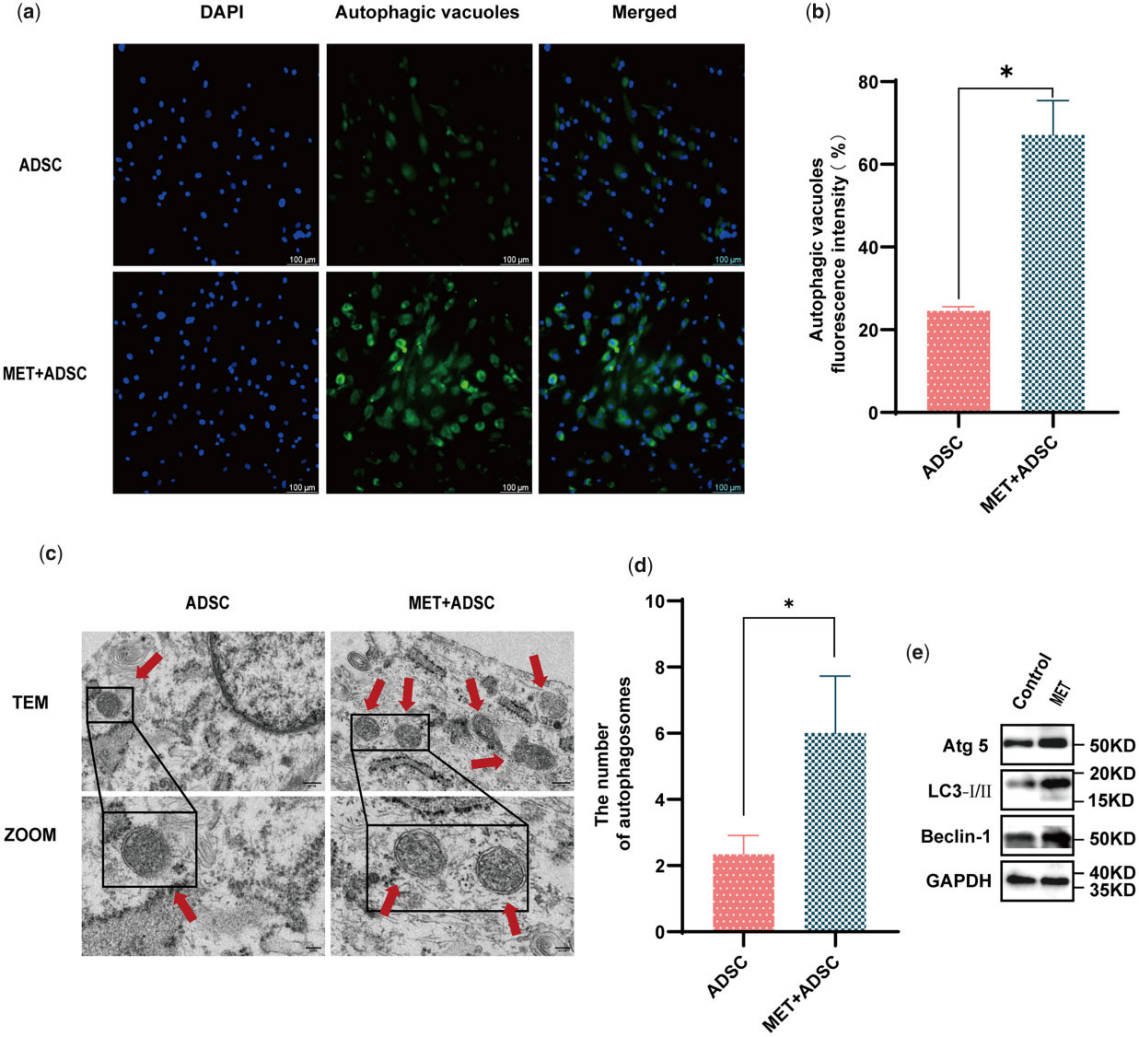
Effects of MET on the autophagy of ADSC *in vitro*. (**a**) Representative images of autophagic vacuoles (green) visualized using immunofluorescence staining (scale bar: 100 μm). (**b**) Autophagic vacuole fluorescence intensity (%) was analysed (*P<0.05, *n*=3). (**c**) Representative TEM images showing the morphology and number of cytolysosomes. Red arrows indicate autophagosomes (scale bar: 100 nm; 200 nm). (**d**) The number of autophagosomes was analysed (*P<0.05, *n*=3). (**e**) Expression of autophagy-related proteins Atg 5, LC3-I/II, and Beclin-1 in untreated and MET-treated ADSC was analysed via western blotting.

As an AMPK activator, MET is closely associated with autophagic activation, and consequently, we examined whether MET can increase the autophagy of ADSC. The findings of autophagy assay revealed that compared with that in untreated ADSC, MET treatment promoted an increase in the number of autophagic vacuoles in ADSC (Fig. 3a and b). On the basis of TEM observations, we established that MET induces autophagosome formation in ADSC (Fig. 3c and d). To determine the levels of autophagy, we examined the protein levels of the microtubule-associated protein light chain 3-II (LC3-II), Autophagy protein 5 (Atg 5), and Beclin-1, which are recognized markers of autophagy, the increased expression of which is typically associated with an increase in autophagic activity. We accordingly found that MET treatment enhanced the expression of these proteins in ADSC (Fig. 3e), which was verified by western blot analysis.

### MET-induced autophagy is associated with VEGFa production and release

**Figure 4.**
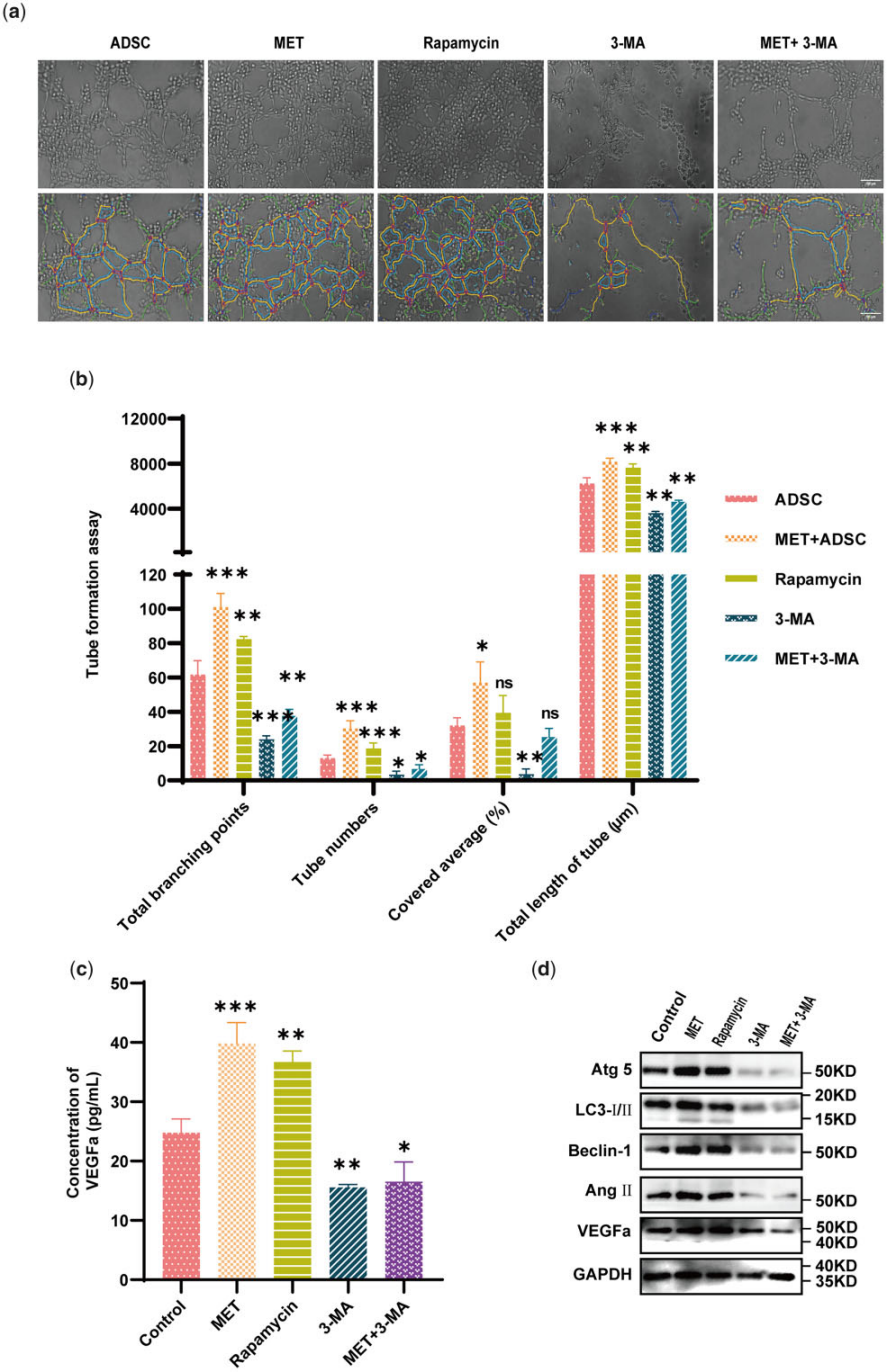
Relationship between MET-induced autophagy and VEGFa production in ADSC. (**a**) Angiogenic differences *in vitro* were evaluated using HUVECs treated with untreated ADSC-CM, MET-treated ADSC-CM, rapamycin-treated ADSC-CM, 3-MA-treated ADSC-CM, or DMEM as the control (scale bar: 100 μm). (**b**) Four indicators were assessed: total branching points, tube numbers, covered area (%), and total length of tubes (μm) (*P<0.05, **P<0.01, ***P<0.001, *n*=3). (**c**) VEGFa secretion in different ADSC-CMs was enhanced in the MET and rapamycin groups but reduced in the 3-MA group, as observed using ELISA (*P<0.05, **P<0.01, ***P<0.001, *n*=3). (**d**) Expression of autophagy-related proteins, Atg 5, LC3-I/II, and Beclin-1, and angiogenesis-related proteins, VEGFa and Ang II, in ADSC was analysed via western blotting.

To assess whether MET-mediated autophagy influences the angiogenic capacity of ADSC, we altered the levels ADSC autophagy using MET, rapamycin (an autophagic activator), and 3-methyladenine (3-MA) (an autophagic inhibitor). The findings of an *in vitro* tube formation assay revealed that ADSC-CM strengthened the HUVECs, and MET mobility and rapamycin significantly enhanced this effect *in vitro* (Fig. 4a). Furthermore, ADSC-CM was found to promote an increase in total branching points, tube numbers, covered area (%), and total length of tubes (μm) of HUVECs in Matrigel. Compared with untreated ADSC, ADSC treated with MET and rapamycin were found to be characterized by promotion of a marked increase in the angiogenic potential of HUVECs (Fig. 4b). An ELISA was used to determine the concentration VEGFa, the results of which revealed a higher release of VEGFa in the MET- and rapamycin-treated cells than in the other cell groups (Fig. 4c). Furthermore, cells in the high autophagy group (MET and rapamycin groups) were characterized significantly higher levels of Ang II and VEGFa protein expression compare with those in the low autophagy group (3-MA and MET + 3-MA groups), as indicated by the findings of western blot analysis (Fig. 4d).

### Autophagy promotes the therapeutic efficacy of ADSC via VEGFa secretion

**Figure 5.**
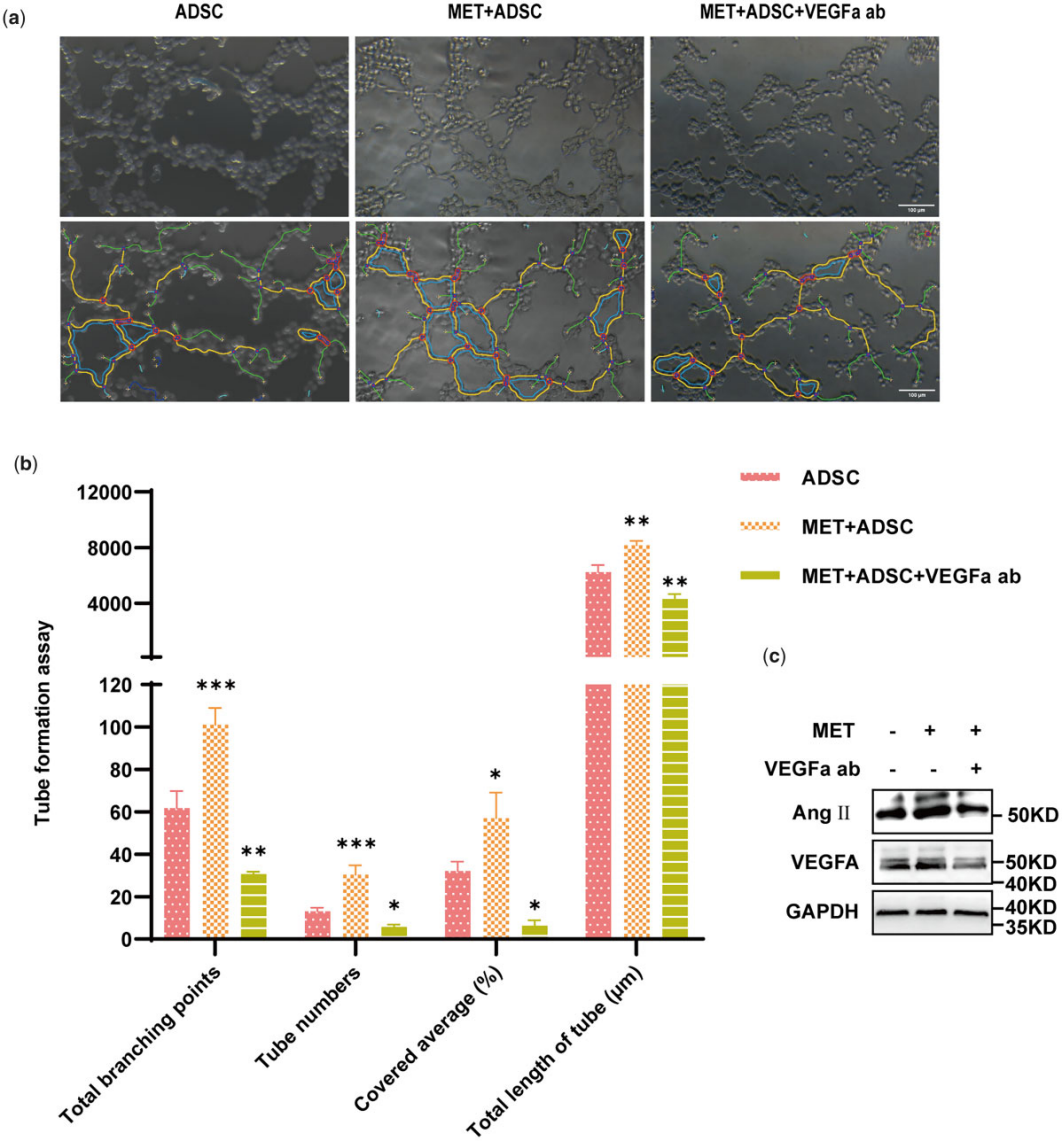
Role of VEGFa secreted by ADSC in the angiogenesis of HUVECs. (**a**) Angiogenic differences *in vitro* were assessed using HUVECs treated with untreated ADSC-CM, MET-treated ADSC-CM, MET + VEGFa antibody (100 ng/ml)-treated ADSC-CM, or DMEM as the control at 6 h (scale bar: 100 μm). (**b**) Four indicators were assessed: total branching points, tube numbers, covered area (%), and total length of tubes (μm) (^ns^*P*>0.05, *P<0.05, **P<0.01, ***P<0.001, *n*=3). (**c**) Expression of the angiogenesis-related proteins VEGFa and Ang II in HUVECs was analysed using western blotting.

To ascertain whether VEGFa secretion contributes to the angiogenesis of HUVECs, a VEGFa antibody was used to neutralize the VEGFa released by ADSC. For this purpose, we used three treatment groups, namely, untreated ADSC-CM (ADSC group), MET-treated ADSC-CM (MET + ADSC group), and MET-treated ADSC-CM with 100 ng/ml VEGFa antibody (MET + ADSC + VEGFa ab group). The different ADSC-CMs were then collected to stimulate HUVECs, after which the HUVECs were collected for western blot and tube formation experiments. The number of tubes formed in the MET + ADSC group was higher than that in the MET + ADSC + VEGFa ab group (Fig. 5a). Moreover, the total branching points, tube numbers, covered area (%), and total length of tubes (μm) were measured using ImageJ software, the results of which indicated that the angiogenic capacity of the MET + ADSC + VEGFa ab group was noticeably inferior to that of the MET + ADSC group (Fig. 5b). Furthermore, we measured the expression of VEGFa and Ang II proteins in HUVECs, and found that compared with the ADSC group, the expression levels of these proteins were higher in the MET + ADSC group and lower in the MET + ADSC + VEGFa ab group, as indicated by the western blot results (Fig. 5c).

### MET can increase the angiogenic capacity of ADSC-CM *in vivo*

**Figure 6.**
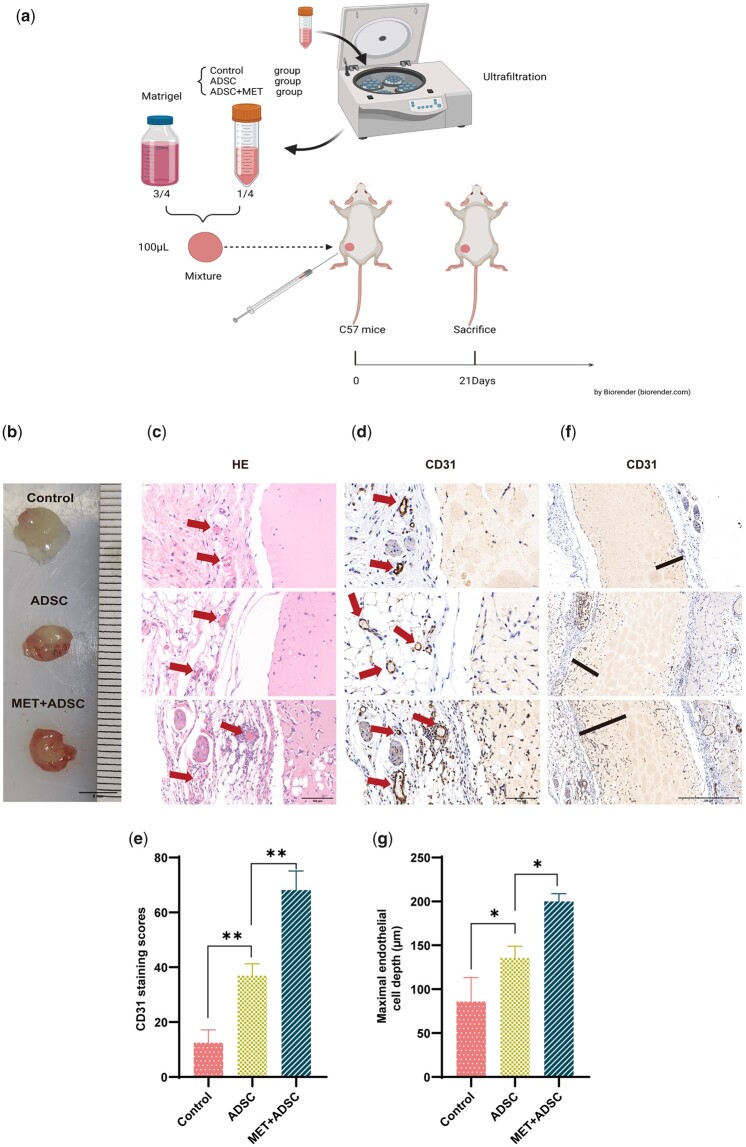
Effects of MET on the angiogenic capacity of ADSC *in vivo*. (**a**) Flowchart of Matrigel plug assay. (**b**) Representative images of Matrigel plugs treated with Human ADSC Complete Medium (control group), untreated ADSC-CM (ADSC group), and MET-treated ADSC-CM (MET + ADSC group) (scale bar: 5 mm). (**c**) H&E staining of Matrigel plugs in the control, ADSC, and MET + ADSC groups (scale bar: 100 μm). Red arrows indicate angiogenesis. (**d**) CD31 immunohistochemistry staining in the control, ADSC, and MET + ADSC groups. Red arrows indicate angiogenesis. (scale bar: 100 μm). (**e**) Quantification of CD31^+^ staining in the control, ADSC, and MET + ADSC group (**P<0.01, *n*=3). (**f**) Depth of ingrowth (black line) of CD31^+^ cells into Matrigel plugs in the control, ADSC, and MET + ADSC groups via immunohistochemistry staining (scale bar: 500 μm). (**g**) Quantification of the depth (µm) of ingrowth of CD31^+^ cells into Matrigel plugs in the control, ADSC, and MET + ADSC groups (*P<0.05, *n*=3).

To further examine the angiogenic activity of the MET-treated ADSC *in vivo*, we mixed Matrigel with different groups of ADSC-CMs. Matrigel mixed with human ADSC-CM was used as the control group, and as treatment groups, we used Matrigel mixed with untreated ADSC-CM (ADSC group) and Matrigel mixed with the MET-treated ADSC-CM (MET + ADSC group). A total of 200 µl of Matrigel solution was subcutaneously injected into the dorsal surface of mice (Fig. 6a). We observed higher microvessel densities in the vicinity of plugs in the MET + ADSC group after 21 days (Fig. 6b), which was confirmed based on H&E staining (Fig. 6c). Furthermore, immunohistochemical staining with CD31 antibody to detect angiogenesis in Matrigel plug sections (Fig. 6d) revealed that the staining scores for CD31 in the MET + ADSC group were 84.6% higher than those in the ADSC group (68.11 versus 36.88, respectively; *P* < 0.01) (Fig. 6e). In addition, we found that the depth of ingrowth of CD31^+^ endothelial cells into the Matrigel plugs was 47.4% greater in the MET + ADSC group than in the ADSC group (199.81 versus 135.60 µm, respectively; *P* < 0.0049) (Fig. 6f and g). These results revealed that angiogenesis was markedly enhanced in the MET-pre-treated ADSC-CM, which was consistent with the data obtained *in vitro* and indicated the significant angiogenic potential of MET-treated ADSC.

## Discussion

Angiogenesis, stimulated by growth factors such as VEGFa, is an essential process for the healing of wounds. ADSC secrete VEGFa, which partially explains the phenomenon whereby the CM of stem cells can promote wound healing. However, given that the variable production of cytokines in ADSC-CM, the clinical application of this medium tends to be limited. To the best of our knowledge, this is the first study to demonstrate how MET enhances autophagy in ADSC, thereby increasing the expression and secretion of VEGFa. On the basis of these findings, we believe that the ADSC-CM derived from MET-treated ADSC may be an effective tool that could be applied to accelerate wound healing by promoting angiogenesis.

MET, the most widely prescribed drug for diabetes, has several effects on stem cells. Zhou *et al.*, for example, reported that MET promotes the osteogenic differentiation of stem cells and reduces oxidative stress [37–39], whereas Alessio *et al.* have demonstrated that MET can inhibit adipogenic differentiation by downregulating the expression of HSP60 [40–42]. Furthermore, Liao *et al.* [36] found that MET can promote the secretion of extracellular vesicles by MSC and retard cell ageing via the autophagy pathway, and found that MET-stimulated MSC show excellent cartilage protection and pain relief in a mouse model of osteoarthritis. In the present study, we found that MET promoted VEGFa secretion in ADSC via autophagy, and demonstrated that MET-pre-treated ADSC showed significant angiogenic capacity both *in vivo* and *in vitro*. These findings indicate that MET promotes angiogenic capacity by increasing the secretion of VEGFa in ADSC.

In this study, we observed that the treatment of ADSC with MET contributed to an increase in the expression of the autophagy-related proteins Atg 5, LC3-II, and Beclin-1, which is consistent with the observed increase in expression of the angiogenesis-related proteins VEGFa and Ang II. In addition, the results of a tube formation assay performed using ELISA revealed that a larger number of tubes were generated in autophagy-enhanced ADSC-CM along with a higher VEGFa expression.

We also found that low MET concentrations (0–1000 μM) had no significant influence on the proliferation of ADSC, which contrasts with previously reported findings [43] indicating that a low MET concentration can promote stem cell proliferation. We speculate that this discrepancy in observations could be ascribed to the differences in cell types used. Nguyen *et al.* [44] found that MET induces stem cell apoptosis, even at a very low concentration (100 μM). Moreover, they established that MET induces AMPK-mediated apoptosis activation by inhibiting the S6K1-Bad-Bcl-xL cell survival signal, thereby promoting the upregulation of apoptosis-related proteins and thus increasing apoptosis in MSC [45]. These findings differ slightly from those obtained in the present study, in which we found that the treatment of stem cells did not influence their proliferation, with the results of a TUNEL assay indicating that MET treatment reduces ADSC apoptosis (Supplementary Fig. S1c).

By performing MSC transplantation for the treatment of myocardial infarction, He *et al.* [45] found that the effect of MSC transplantation in rats simultaneously administered oral MET was not as pronounced as that observed in response to stem cell transplantation alone, resulting in an increase in the extent of myocardial infarction and limiting the recovery of cardiac function. In a further study on diabetic cardiomyopathy, Ammar *et al.* [46] found that bone marrow mesenchymal stem cell (BMSC) transplantation can prevent the fibrosis of diabetic hearts and promote angiogenesis. However, when rats were orally administered MET, these authors detected a reduction in the BMSC-mediated cardiac protection. Ammar *et al.* also established that BMSC and MET can enhance cardiac function when used alone, although they detected no synergistic effect on heart protection when these were applied in combination. In addition, they found that MET-mediated AMPK activation led to the abnormal homing and low survival of BMSC in the hearts of diabetic patients [46]. The results of these studies contrast with those obtained in the present study, in which we demonstrated that MET can promote stem cell angiogenesis. In this regard, however, these authors used different treatment approaches, in which rats were administered MET orally, and the objective was to investigate whether MET and stem cells have a synergistic cardio-protective effect. To date, the mechanisms underlying the effects orally and locally administered MET have yet been fully clarified. In this study, the ADSC-CM we used was extracted following pre-treatment with MET, and consequently, MET would directly influence other cells *in vivo*. We focussed primarily on changes in the secretory capacities of ADSC in response MET stimulation. In addition, it has previously been established that MET can promote cellular autophagy [26]. Our findings in this study similarly confirmed that the autophagy of ADSC was enhanced, and those of previous research have indicated that the activation of autophagy in stem cells can contribute to reducing the levels of apoptosis [47]. However, these findings tend to contrast with the aforementioned experimental results and therefore warrant further study.

## Conclusion

In conclusion, our findings in this study revealed that ADSC pre-treated with MET are characterized by a more pronounced secretion of VEGFa and enhanced angiogenic capacity *in vivo* and *in vitro*, and we demonstrate that this process is mediated via MET-induced autophagy.

## Supplementary Material

rbad043_Supplementary_Data

## Data Availability

No underlying data were collected or produced in this study.
