# Supplementary material for: Metformin promotes angiogenesis by enhancing VEGFa secretion by adipose-derived stem cells via the autophagy pathway
Source: Regen Biomater. 2023 Apr 24;10:rbad043. doi: 10.1093/rb/rbad043 (PMC10224801; doi:10.1093/rb/rbad043)
Supplement: rbad043_Supplementary_Data [file rbad043_supplementary_data.docx]

**Supplementary Figure**


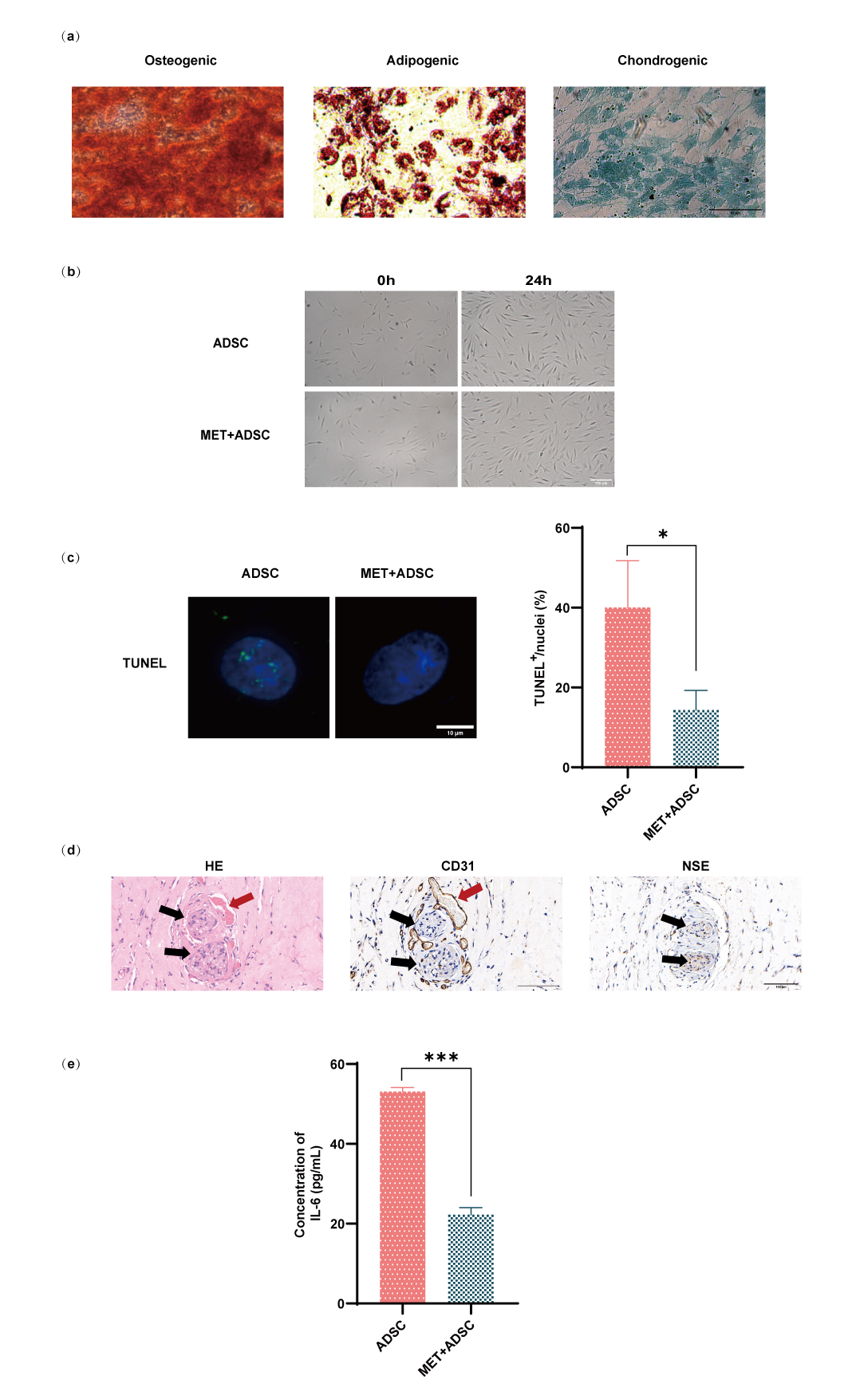


**Supplementary Figure 1**

(**a**) Representative photographs of Alizarin Red S, Oil Red O, and Alcian Blue staining of induced ADSC (scale bar: 50 μm); (**b**) Analysis of stem cell morphology. An optical microscope was used to examine the appearance of untreated and MET-treated ADSC (scale bar: 200 μm); (**c**) TUNEL assay results revealed that treatment with MET decreased the level of apoptosis in ADSC (scale bar: 10 μm), and the percentage of TUNEL^+^/nuclei (%) were analysed (*P < 0.05, n = 3); (**d**) Representative photographs of H&E, CD31, and NSE staining in Matrigel plugs of the MET+ADSC group (scale bar: 100 μm). Black arrows indicate nerve regeneration, and red arrows indicate blood vessel regeneration; (**e**) The secretion of IL-6 was tested in non-treated ADSC-CM and MET-treated ADSC-CM by ELISA (***P<0.001, n=3).
